# Chronic widespread musculoskeletal pain, fatigue, depression and disordered sleep in chronic post-SARS syndrome; a case-controlled study

**DOI:** 10.1186/1471-2377-11-37

**Published:** 2011-03-24

**Authors:** Harvey Moldofsky, John Patcai

**Affiliations:** 1 Sleep Disorders Clinic of the Centre for Sleep and Chronobiology, 340 College St., Suite 580, Toronto, ON M5T 3A9, Canada; 2 St. John's Rehab Hospital, 285 Cummer Ave, Toronto, ON M2M 2G1, Canada; 3 Department of Medicine, University of Toronto, #3S805 - 200 Elizabeth Street, Toronto, ON M5G 2C4 Canada

## Abstract

**Background:**

The long term adverse effects of Severe Acute Respiratory Syndrome (SARS), a viral disease, are poorly understood.

**Methods:**

Sleep physiology, somatic and mood symptoms of 22 Toronto subjects, 21 of whom were healthcare workers, (19 females, 3 males, mean age 46.29 yrs.+/- 11.02) who remained unable to return to their former occupation (mean 19.8 months, range: 13 to 36 months following SARS) were compared to 7 healthy female subjects. Because of their clinical similarities to patients with fibromyalgia syndrome (FMS) these post-SARS subjects were similarly compared to 21 drug free female patients, (mean age 42.4 +/- 11.8 yrs.) who fulfilled criteria for fibromyalgia.

**Results:**

Chronic post-SARS is characterized by persistent fatigue, diffuse myalgia, weakness, depression, and nonrestorative sleep with associated REM-related apneas/hypopneas, an elevated sleep EEG cyclical alternating pattern, and alpha EEG sleep anomaly. Post- SARS patients had symptoms of pre and post-sleep fatigue and post sleep sleepiness that were similar to the symptoms of patients with FMS, and similar to symptoms of patients with chronic fatigue syndrome. Both post-SARS and FMS groups had sleep instability as indicated by the high sleep EEG cyclical alternating pattern rate. The post-SARS group had a lower rating of the alpha EEG sleep anomaly as compared to the FMS patients. The post-SARS group also reported less pre-sleep and post-sleep musculoskeletal pain symptoms.

**Conclusions:**

The clinical and sleep features of chronic post-SARS form a syndrome of chronic fatigue, pain, weakness, depression and sleep disturbance, which overlaps with the clinical and sleep features of FMS and chronic fatigue syndrome.

## Background

In light of public health concerns about the adverse effects of the recent H1N1 pandemic viral infection, it is noted that the long-term effects on survivors of those who survive severe illness are unknown. In this paper we report the results of our study of the long term adverse effects of Severe Acute Respiratory Syndrome (SARS) that emerged from South East Asia in early 2003 as the first contemporary novel severe acute infectious global health problem. In North America, Toronto experienced the bulk of cases that largely affected health care workers[1]. The Ontario health authorities alerted health care personnel on March 14, 2003 about 4 family members with atypical pneumonia that resulted in two deaths. A province-wide emergency was declared on March 26^th^, 2003 when it became evident that these cases were the epidemiological link to SARS. The government and health care providers took steps to contain the spread of SARS by enacting infection control procedures, screening and isolating those people who were exposed, and admitting affected personnel, many of whom were health care workers, to special hospital SARS units. The public health precautions proved effective enough that by June 12, 2003 there were no more new cases. During those 3 months 273 people were identified as being confirmed SARS [2] cases. 44 [3] died. Because identification of victims and containment were the orders of the time, medical attention focused upon the features of the acute phase of the illness. They were identified as having new onset of fever, documented elevated temperature, and were likely to have nonproductive cough, myalgia, and dyspnea. Such individuals may have been exposed to patients who had traveled to a location known to harbour such cases, i.e., South China, South East Asia, or may have acquired these symptoms as the result of direct contact or exposure [1,4]. Subsequently a novel coronavirus was identified as the cause of the acute outbreak [5-7].

Although the epidemic in Toronto was considered controlled because no new cases appeared after June 2003 with no one remaining in quarantine [1,8] one year later when life in the hospitals and city had returned to normal, a cohort of post-SARS patients remained disabled and unable to return to their work. They complained of persisting debilitating physical symptoms including variable musculoskeletal pain, profound weakness, easy fatigability, shortness of breath that accompanied psychological distress and major sleep problems. Because of the possibility of persistence of sleep-related respiratory dysfunction and arousal disturbances in the sleep EEG that could affect daytime fatigue and mood disturbances in the patients with the greatest clinical sleep disturbances, we examined their sleep physiology and coincident somatic and psychological symptoms. This is the first report of the long-term adverse effects of SARS on sleep and somatic symptoms.

## Methods

The University Health Network human research ethics board approved the retrospective study of clinically necessary sleep studies, and signed informed consent forms were obtained from all patients. 19 females (mean age 46.29 +/- 11.02y., BMI = 28.26 +/- 6.88) and 3 males, (all except one being health care workers) were assessed about 19.8 months after onset of the acute SARS illness (range: 13 to 36 months) with hospitalization and/or quarantine for SARS. They were part of a cohort of 50 post-SARS patients who had come under the care of the Ontario Workers' Safety and Insurance Board (WSIB) because of persistent impaired health that interfered with their hospital work capabilities. They had been sent by WSIB for a complex, intense, interdisciplinary clinical program of physical and psychological restorative rehabilitation. This post-SARS group was compared to a small group of younger healthy 8 females (mean age 30 +/- 6.7 y., p = 0.0002), but with a similar BMI (24.8 +/- 6.1, p =.242) by employing standard self-ratings of physical symptoms [the Wahler Physical Symptom Inventory (WPSI)] [9], of depression [Beck Depression Inventory (BDI)] [10,11], of self rated symptoms of post traumatic stress disorder (PTSD) because of their exposure to the threat of death as the result of their exposure to the SARS virus[PTSD check list civilian version {PCL-C}] [12], and of sleep symptoms [the Sleep Assessment Questionnaire^c ^(SAQ^c^)] [13]. The SAQ is a 17-item questionnaire that has been found to be useful for identifying sleep disorders related to chronic fatigue. One overnight polysomnography was employed in order to objectively evaluate sleep physiology. The procedures included electroencephalogram (EEG C3, C4) electro-oculogram, submental and bilateral anterior tibialis electromyogram, single anterior lead electrocardiogram, measures of respiration comprising measures of airflow with oral-nasal thermistors and respiratory impedance plethysomography, and pulse oximetry. An experienced registered polysomnographic technologist completed blind ratings of sleep physiological indices [14] and standardized ratings (1 to 5) of the presence of a measure of arousal in the non rapid eye movement sleep EEG (NonREM sleep), known as the alpha frequency anomaly [15] where 1 was the percentage of alpha EEG in Non REM sleep (7.5 Hz to 12Hz) less than 20% and where 5 was more than 80% of alpha EEG in Non REM sleep. In order to evaluate daytime lethargy the overnight sleep study was followed by the multiple sleep latency test (MSLT) comprising of at least four 20 minute nap opportunities at 2 hour interval beginning 2 hours after morning awakening [16]. Self ratings of symptoms pre-sleep and post sleep consisted of standard measures of total regional musculoskeletal pain severity (0-24), of fatigue (1-7) [17], and sleepiness [18] that had been used in previous studies of patients with fibromyalgia syndrome (FMS) and chronic fatigue syndrome (CFS). Because clinically the post-SARS patients described many of the features seen in patients with FMS we also compared the self ratings of pain, fatigue and sleepiness symptoms and objective indices of overnight sleep physiology to an aged matched population of 21 drug free female patients, (mean age 42.4 +/- 11.8 yrs.) who fulfilled criteria for fibromyalgia [19]. They had participated in a double blind placebo- controlled drug trial, to which they had provided their ethics approved signed consent. Only the initial overnight pre- treatment sleep study was employed for the purposes of comparison with this post-SARS chronically ill patient population. In addition to the standard sleep physiological indices described above we analyzed another sleep EEG anomaly known as a high frequency of cyclical alternating pattern (CAP). The CAP rate has been found to be a quantified measure of EEG sleep stability where frequent periodic EEG arousal disturbances indicate sleep instability or poor quality sleep. This high frequency of CAP has been related to less efficient sleep and the severity of symptoms of patients with FMS[20,21]. Objective ratings of CAP rate was assessed using a validated, computerized automatic detection methodology (Somnologica) [22,23]. Statistical analyses were completed between group 2 tail t-tests for behavioural self-ratings, sleep physiological indices, and pre-post sleep ratings of current symptoms. Bonferroni corrections were performed on the multiple t tests.

## Results

In comparison to healthy subjects, post-SARS subjects reported more physical symptoms on the WPSI (mean 10.6 +/- 5.0 vs. 0.4 +/- 0.5, p <.0001). On most days they complained of tiredness, difficulty sleeping, myalgia and muscular weakness. They had more mild to moderate depressive symptoms (BDI mean = 13.3 +/- 8 vs.0.86 +/- 1.5, p < .0001), more sleep disturbances on the SAQ^c ^(mean total score = 30.9 +/- 5 vs. 10.9 +/- 3.4, p < .0001), more fatigue post-sleep (p <.05), and more myalgia pre- and post-sleep (p < .01). See Table 1. For the 21 patients that completed the PCL-C, the mean score was 40.75 +/- 10.26. Two patients had scores of 50 or more that would be suggestive of the symptoms seen in patients with PTSD [24].

**Table 1:** Sleep in SARS vs. Healthy Controls

Sleep Parameter	SARS	Healthy Controls	Significance
	**Mean (SD)**	**Mean(SD)**	**P**

Sleep onset latency ( min.)	24.13 (21.63)	29.34(31.48)	n.s.

Total sleep time (min)	370.83 (83.84)	343.43(38.47)	n.s.

Sleep Efficiency %	77.44 (13.56)	83.23( 12.36)	n,s.

Stage 1%	9.11 (4.13)	6.60(4.61)	n.s.

Stage 2%	60.22 (9.95)	49.77(6.53)	p = 0.006

Stage 3%	7.83 (6.36)	4.84(2.11)	n.s.

Stage 4%	6.27 (5.80)	9.70 (2.84)	p = 0.051.

REM onset Latency ( min.)	136.79 (63.72)	87.23 (38.47)	p = 0.004

REM %	16.57 (5.94)	17.27 (5.35)	n.s.

Apnea/Apnea-Hypopneas Index (no. per hr.of sleep)	4.70 (5.53)	2.73 (2.08)	n.s.

REM Apnea-Hypopnea Index (no. per hr.of REM sleep)	14.33 (15.41)	N/A	N/A

NREM 02 saturation (max/min)	99.13 (0.68) 92.09 (3.61)	N/A	N/A

REM 02 saturation (max/min)	98.69 (0.93)/92.24 (4.60)	N/A 96.50 (0.71)	N/A N/A

The overnight sleep physiology in the post-SARS group showed more arousal disturbances and the alpha EEG sleep disorder which consists of the anomalous appearance of the EEG alpha frequency (7.5-12 Hz) in approximately 50% of sleep. There was more stage 2 NonREM sleep and a delay in onset to REM sleep, but no other significant differences in measures of sleep EEG. See Table 1.

In measures of sleep-related respiratory disturbances, 5 post-SARS subjects who snored had variable daytime sleepiness on the MSLT (defined as rapid onset to sleep in less than 8 min on at least one of the 4 or 5 nap opportunities, range from 3-8 min). Two of these Post-SARS subjects (ages 63 yrs., BMI = 28, and 49 yrs, BMI = 32) who were being treated for hypertension had moderate and mild sleep hypopnea/apnea disorder (with a respiratory distress index (RDI) = 18.8 and 8.4 respectively) and arterial oxygen desaturation (minimum of 81.2% and 83% respectively). The only other person (age 57 yrs, BMI 25.5) being treated for hypertension had very mild elevation of RDI (7.5) but no snoring, no significant sleep-related arterial oxygen desaturations or daytime sleepiness. The others had no specific disturbances in sleep-related respiration. The limited number of healthy subjects and their incomplete detailed data on REM and NonRem arterial blood oxygen saturations did not permit proper comparison with such data from the post-SARS group.

Although most complained of being depressed only two were receiving antidepressants (citalopram), one of which had sleep apnea. All patients were seen by clinical psychologists unless they declined to do so, and psychiatric consultation was available to all if requested by the patient or other team members.

In the comparison between the post-SARS & FMS patients there were no clinically significant sleep related breathing disturbances or periodic leg movements during sleep. Both groups had a similarly elevated measure of sleep instability as indicated by the high cyclical alternating pattern rate [22,23], compared to the published norms as reported by Parrino [25] et al. These are also comparable to the findings of Rizzi et al in their study of CAP in FM patients.

FMS patients, however, showed a significantly higher rating of the alpha EEG sleep anomaly than did the post-SARS patients. Other than there being more NonREM stage 2 sleep and a relative delay in onset to the initial REM sleep episode there were no other differences in their stages of sleep EEG. In their ratings of somatic symptoms post-SARS patients reported pre and post sleep fatigue and sleepiness similar to that reported by patients with FMS. Unlike the FMS patients they complained of less pre sleep and post sleep musculoskeletal pain symptoms (see table 2). See Table 2.

**Table 2:** Sleep, Pain and Fatigue in SARS vs. FMS Subjects

Sleep Parameter	SARS (n = 22)	Fibromyalgia (n = 21)	Significance
	**Mean (SD)**	**Mean (SD)**	

Sleep onset latency ( min.)	24.13 (21.63)	18.37 (35.39)	n.s.

Total sleep time (min)	370.83 (83.84)	338.54 (76.26)	n.s.

Sleep Efficiency %	77.44 (13.56)	79.34 (15.63)	n.s.

Stage 1%	9.11 (4.13)	9.76 (3.66)	n.s.

Stage 2%	60.22 (9.95)	54.61(5.41)	0.031

Stage 3%	7.83 (6.36)	7.35 (3.08)	n.s.

Stage 4%	6.27 (5.80)	9.53 (6.18)	n.s.

REM onset Latency ( min.)	136.79 (63.72)	87.26 (35.78)	0.004

REM %	16.57 (5.94)	18.77 (4.81)	n.s.

Apnea/Hypopneas Index (no. per hr.of sleep)	4.70 (5.53)	3.29 (2.37)	n.s.

Periodic leg movements ( no.per hr of sleep)	2.03 (5.64)	2.38 (3.81)	n.s.

Arousals per hr of sleep	14.01 (7.59)	11.31 (5.31)	n.s.

CAP rate per hr of sleep	71.64 ()(14.25)	70.39 (15.64)	n.s.

Alpha EEG sleep (1-5)	3.00 (0.63)	3.50(0.61)	0.014

Presleep Pain Presleep Fatigue (1-7)	6.24 (4.01) 4.57 (1.57)	10.95 (5.74) 4.30 (1.08)	0.005 n.s.

Presleep Sleepiness (1-7)	2.76 (1.14)	4.30 (1.08)	0.0001

Post Sleep Pain (0-24)	7.10 (3.81)	11.75 (6.45)	0.009

Post Sleep Fatigue (1-7)	4.30 (1.87)	4.60 (1.23)	n.s.

Post Sleep Sleepiness (1-7)	3.45 (1.57)	3.90 (1.12)	n.s.

## Discussion

This is the first report of the long-term adverse effects of widespread chronic pain, fatigue, psychological distress and disturbed sleep after acute SARS that contributed to the failure to return to productive work of a small cohort of health care professionals at least one year after their acute illness. After their initial SARS symptoms of severe respiratory distress, fever with evidence for infiltrates in their lungs had remitted so that they were considered to be no longer in need of special quarantine and acute treatment measures, a constellation of symptoms persisted that interfered with their ability to function in their occupations. This was then a potentially biased selection process of 22 patients who were members of a group of 50 chronically ill survivors of SARS. A larger study of 107 patients from Toronto, with a more widely selected population [26] had shown that, at the one year mark some continued to describe problems with pain, reduced vitality, physical, mental, and social functioning. Only 14 (13%) were asymptomatic, leaving 93 patients (87%) symptomatic, where 18 (17%) had not returned to work, and 10 (9%) had returned to modified work. If one presumes that the asymptomatic group was most likely to return to unmodified work, then of the 79 patients returning to unmodified work, only 14 were asymptomatic. This leads to the arithmetic conclusion that 65 (82%) of their patients who returned to unmodified work were nevertheless continuing to work despite ongoing symptoms.

Furthermore, 5 of our 22 subjects showed variable daytime sleepiness, which was associated with snoring indicative of sleep disordered breathing, but not necessarily overt sleep apnea. While sleep-related breathing disturbances have been reported in some patients with FMS/CFS [27-29] only three post-SARS subjects exhibited mild to moderate sleep apnea/hypopneas. However, they may have had this sleep disorder before SARS because they were being treated for hypertension, a common causal risk for untreated sleep apnea [30]. Nevertheless, these sleep-related respiratory disturbances together with the alpha EEG sleep disorder may have contributed to their unrefreshing sleep and daytime symptoms.

Our single overnight study showed sleep physiological changes in stage 2 and REM onset latency that may have been influenced by being a feature of the potential adverse effect on sleep of the procedures employed in the study, known as the first night effect. The groups, however, were compared under similar circumstances although the SARS healthcare subjects may have been more sensitive to being tested in the sleep laboratory, and being more depressed. The two who were receiving antidepressant medications (citalopram) did not differ in any of the sleep parameters from the others who were equally depressed and not receiving such medications.

In our cohort, their disabling chronic fatigue, variable nonspecific myalgia, depression and sleep disturbances are similar to those experienced by patients with post-febrile Chronic Fatigue Syndrome (CFS) [31] and Fibromyalgia Syndrome (FMS) [32]. Indeed, physiological changes in their sleep EEG, i.e., the alpha EEG sleep anomaly is a common feature in such patients who commonly complain of unrefreshing sleep, fatigue, musculoskeletal pain, impaired cognitive functioning, and emotional distress [21,23,33-36]. In the comparison of the post-SARS patients to the FMS subjects we noted similar sleep EEG elevated cyclical alternating pattern rate as previously reported by ourselves and others [22,23]. However, in this study both the alpha EEG sleep ratings and the pain ratings were greater in FMS disorder than the post-SARS patients. Indeed the post-SARS patients seem to be similar to CFS patients where their focus is on fatigue symptoms rather than the pain.

Some contribution to the post-SARS persistent sleep, pain, fatigue, and depressive symptoms may have occurred as the result of the psychologically traumatic effects of their acute infectious illness. That is, these symptoms may have arisen as the result of their isolation from family and friends, uncertain outcome and threat of death. While as a group the post-SARS patients did rate themselves as having psychological distress only 2 patients described features on the PCL-C rating scale that are attributed to those with PTSD [12]. Such distressing experiences together with their acute SARS may have contributed to their alpha EEG arousal disturbances in sleep, recurrent nightmares and their inability to obtain restful sleep. Sleep difficulties have been reported in health care workers who did not have SARS themselves, but who did care for patients with SARS [37]. Indeed, similar unrefreshing sleep, fatigue and musculoskeletal pain symptoms occur in healthy people who have been experimentally exposed to several nights of frequent noise induced disruption of slow wave sleep, which artificially produces the periods of the alpha EEG sleep [38].

In addition there is the possibility that the sleep disorder, fatigue and behavioral symptoms may have occurred as the result of the Coronavirus A directly; this virus is known to invade the central nervous system and peripheral tissues [39,40]. Viral particles and viral genome sequences were isolated from the cytoplasm of neurons more commonly in the hypothalamus and the cortex [41,42]. Although the evidence indicates that the virus crosses the blood-brain barrier into the brain, the route of infection in humans remains unknown [43]. In mice that are transgenic for the SARS-CoV receptor (human angiotensin-converting enzyme 2) the virus enters the brain primarily via the olfactory bulb. Thereafter the infection spreads rapidly via neurons throughout the brain [44]. The virus may have resulted in chronic post-inflammatory CNS pathology that adversely affects sleep, pain sensitivity, and energy. In previous studies of chronic post-viral fatigue, both severity of the initial illness [45] and symptom-attributional style and physician behaviour [46] have been associated with such outcomes.

The literature regarding SARS has documented many physical and psychological sequelae of the illness in both the short and the long term. Many studies did not note sleep disturbances, most likely on the basis that the focus of the study was elsewhere, even when one would expect sleep disturbances - such as when reporting incidence of post traumatic stress disorder (PTSD). In the studies where questions were asked about sleep, sleep disturbances were noted, although prevalence numbers are hard to find (table 3).

**Table 3:** Published Studies

re Patients Post-SARS (re Sleep)		
**Location**	**Fol*l*owup Time**	**Outcomes**

Toronto	Acute illness (Retrospective)	no discussion sleep [2]

	21 day	no discussion of sleep [1]

	3 weeks post discharge	Appears to be 100% prevalence insomnia (two cases were severe) [4]

	One year	complaints of sleep disturbance averaging 47% prevalence [26]

Asia	"peak of outbreak"	Sleep problems noted, no incidence given [50]

	6 months	No discussion sleep [51]

	Summary 2005	No discussion sleep [52]

	Summary 2006	No discussion sleep [53]

**re Health Care Workers Not Ill With SARS (re Sleep)**		

Toronto	4 weeks	No prevalence given, but "sleep may be the first casualty"[54]

	~ 5 months	"Stigma, fear, frustration" but no discussion sleep [55]

	26 months	Negative psychological effects but no discussion sleep [56]

Asia	5 weeks	No discussion sleep [57]

	April, May 2003	No discussion sleep [58]

	3 months	Sleep "poor", improved by prevention program [37]

	30 months	"Mental health catastrophe" but no discussion sleep [59]

## Conclusions

Chronic post-SARS is characterized by persistent fatigue, diffuse myalgia, weakness, depression, and nonrestorative sleep with associated REM-related apneas/hypopneas and alpha EEG sleep disorder. These clinical and sleep features of chronic post-SARS are similar to those features which may be found in patients with chronic fatigue syndrome/fibromyalgia. This report of the possible contribution of the coronaviral SARS to the emergence of chronic fatigue, unrefreshing sleep fatigue and widespread musculoskeletal pain symptoms also raises the question of the specificity the infectious retrovirus, XMRV, in blood cells that was recently reported [47] but is now a source of controversy as to its significance and specificity for patients with chronic fatigue syndrome [48,49]. A longer term, large scale study is needed to establish the contribution of epidemic and pandemic viral disease to the disordered sleep, chronic fatigue and somatic symptoms of chronic fatigue/fibromyalgia syndrome.

## Competing interests

For Dr. Patcai - nil. Dr. Moldofsky is a consultant to, or is on the advisory boards of, or has received speakers honoraria, and/or research support from Eli Lilly, Jazz, Tonix (Krele), Lundbeck, Merck, Pfizer, Pierre Fabre, Sanofi Aventis, Schering Plough and Valeant.

## Authors' contributions

Both authors had a physician-patient relationship with all patients in this study, and clinically reviewed the data for each patient as the sleep studies were done. HM did the initial data analysis, which was then reviewed by both authors. The first draft was written primarily by HM with some sections by JP, and extensively discussed, re-worked and edited by both authors. Reference contributions were from both. Both authors read and approved the final draft.

## Pre-publication history

The pre-publication history for this paper can be accessed here:

http://www.biomedcentral.com/1471-2377/11/37/prepub
